# Lipid Remodeling Reveals the Adaptations of a Marine Diatom to Ocean Acidification

**DOI:** 10.3389/fmicb.2021.748445

**Published:** 2021-10-14

**Authors:** Peng Jin, Zhe Liang, Hua Lu, Jinmei Pan, Peiyuan Li, Quanting Huang, Yingyan Guo, Jiahui Zhong, Futian Li, Jiaofeng Wan, Sebastian Overmans, Jianrong Xia

**Affiliations:** ^1^School of Environmental Science and Engineering, Guangzhou University, Guangzhou, China; ^2^Jiangsu Key Laboratory of Marine Bioresources and Environment, Jiangsu Ocean University, Lianyungang, China; ^3^King Abdullah University of Science and Technology (KAUST), Biological and Environmental Sciences and Engineering Division (BESE), Thuwal, Saudi Arabia

**Keywords:** food quality, adaptation, ocean acidification, lipidomics, diatoms

## Abstract

Ocean acidification is recognized as a major anthropogenic perturbation of the modern ocean. While extensive studies have been carried out to explore the short-term physiological responses of phytoplankton to ocean acidification, little is known about their lipidomic responses after a long-term ocean acidification adaptation. Here we perform the lipidomic analysis of a marine diatom *Phaeodactylum tricornutum* following long-term (∼400 days) selection to ocean acidification conditions. We identified a total of 476 lipid metabolites in long-term high CO_2_ (i.e., ocean acidification condition) and low CO_2_ (i.e., ambient condition) selected *P. tricornutum* cells. Our results further show that long-term high CO_2_ selection triggered substantial changes in lipid metabolites by down- and up-regulating 33 and 42 lipid metabolites. While monogalactosyldiacylglycerol (MGDG) was significantly down-regulated in the long-term high CO_2_ selected conditions, the majority (∼80%) of phosphatidylglycerol (PG) was up-regulated. The tightly coupled regulations (positively or negatively correlated) of significantly regulated lipid metabolites suggest that the lipid remodeling is an organismal adaptation strategy of marine diatoms to ongoing ocean acidification. Since the composition and content of lipids are crucial for marine food quality, and these changes can be transferred to high trophic levels, our results highlight the importance of determining the long-term adaptation of lipids in marine producers in predicting the ecological consequences of climate change.

## Introduction

Diatoms are responsible for ∼20% of global primary production and play crucial roles in carbon and silicon biogeochemical cycles (Field et al., 1998). Their fixed carbon is partitioned into either carbohydrates or lipids (Kroth et al., 2008). The lipids of diatoms consist of almost all lipid classes, including both polar lipids (Guschina and Harwood, 2006) and non-polar lipids [free fatty acids, sterols, glycerols and especially triacylglycerols (TAGs)]. TAGs of diatoms have been increasingly studied for their potential as nutritional sources and for biofuel production (Hildebrand et al., 2012; Shahidi and Ambigaipalan, 2018). Fatty acid profiles of diatoms are enriched with medium-chain and very long-chain polyunsaturated fatty acids (PUFAs), namely ω3 fatty acids such as eicosapentaenoic acid (EPA) (Dunstan et al., 1993; Guschina and Harwood, 2006). In aquatic food webs, PUFAs are exclusively synthesized by phytoplankton and cannot be synthesized *de novo* by metazoans, and therefore must be acquired by non-phytoplanktonic organisms *via* their diet (Hixson et al., 2015). As such, PUFAs are important determinants of food quality and, consequently, an important indicator for the health and optimal functioning of marine and freshwater food webs (Dalsgaard et al., 2003). The polar lipid fraction of diatoms mainly consists of digalactosyldiacylglycerol (DGDG), monogalactosyldiacylglycerol (MGDG), sulfoquino vosyldiacylglycerol (SQDG), phosphatidylinositol (PI), phosphatidylglycerol (PG), phosphatidylcholine (PC), and minor lipids such as betaine lipids (Stonik and Stonik, 2015).

Global change induces many alterations in marine environments, such as ocean acidification (Gattuso et al., 2015). The molecular, physiological, biochemical and ecological responses of diatoms to ocean acidification have been studied extensively in the last two decades (Gao et al., 2012; Hennon et al., 2015; Petrou et al., 2019; Shi et al., 2019; Li et al., 2021; see also comprehensive reviews by Gao and Campbell, 2014; Bach and Taucher, 2019 and references therein). It has been reported that the lipid contents and fatty acid compositions of diatoms are highly dependent on CO_2_ concentrations (Bermúdez et al., 2015; Wang et al., 2017; Abreu et al., 2020). For example, ocean acidification decreased the content of PUFAs in the marine diatom *Cylindrotheca fusiformis* by ∼3% (Bermúdez et al., 2015). The mechanisms through which CO_2_ affects the composition of fatty acids in microalgae are still unclear, however, it has been suggested that elevated CO_2_ can enhance the synthesis and accumulation of saturated fatty acids (Sato et al., 2003). This response reduces cell membrane fluidity, which helps the organism to cope with pH reductions and facilitates the regulation of cell homeostasis (Lane and Burris, 1981; Rossoll et al., 2012). More recently, it has been reported that the operation of carbon concentration mechanisms (CCMs) of microalgae are tightly coupled with their lipid metabolisms (Renberg et al., 2010; Abreu et al., 2020) and that the CCMs of diatoms are partially down-regulated under ocean acidification conditions (Trimborn et al., 2009; Wu et al., 2010; Hopkinson et al., 2011; Yang and Gao, 2012). The down-regulations of CCMs led to decreased photorespiration and symptoms of oxidative stress because of an increase in the electron sink constituted by CO_2_ fixation (Raven, 2010; Renberg et al., 2010). These changes consequently altered the composition of lipids (such as DGDG, MGDG, and triacylglycerol, given their importance for cell functioning, especially in stress-responses (e.g., oxidative stress) (Bréhélin et al., 2007; Abreu et al., 2020). However, most published studies have analyzed responses of lipid metabolisms in the short term only (i.e., 1–2 weeks), and were thus unable to resolve long-term responses to ocean acidification conditions. Since diatoms are characterized by large population sizes, standing genetic variations and short generation times (Reusch and Boyd, 2013; Collins et al., 2020), they have a high potential to adapt to acidifying oceans, as recently indicated (Li et al., 2017; Zhong et al., 2021).

To address this fundamental knowledge gap, we carried out a ∼400 days selection experiment with the model marine diatom *Phaeodactylum tricornutum*, and employed a lipidomics approach to investigate long-term responses in the lipid metabolism of diatoms to different emission scenarios associated with ocean acidification.

## Materials and Methods

### Culture Conditions

Cultures of *Phaeodactylum tricornutum* Bohlin bac-2, obtained from the Institute of Oceanology at the Chinese Academy of Sciences, were maintained in half-strength Guillard’s “F” solution (Guillard and Ryther, 1962). Prior to the long-term experiments, the cultures were kept in 15°C plant growth chambers (HP1000G-D, Ruihua) under a photon flux of 100 μmol photons m^–2^ s^–1^ with a light:dark cycle of 12 h:12 h (HP1000G-D, Ruihua). To initiate the long-term selection experiments, the single-clone cultures were diluted into triplicates (500 mL each) and grown at low CO_2_ (400 μatm, ambient CO_2_ condition, denoted LC) and high CO_2_ (1,000 μatm, projected year 2100 high CO_2_ according to the high emission scenario RCP 8.5, IPCC, 2014, denoted HC) levels. The LC condition was attained by pre-aerating the medium with the ambient outdoor air, while the HC treatment was achieved within a plant growth chamber (HP1000G-D, Ruihua). In the chamber, the target CO_2_ level of 1,000 μatm was obtained by mixing air and pure CO_2_ gas. The CO_2_ partial pressure was continuously monitored and maintained at 1,000 ± 50 μatm. Triplicate semi-continuous batch cultures were grown for ∼400 days under the two selection regimes (i.e., LC, HC). After the 400-days selection period, the cells have grown for approximately 885 and 883 generations under LC and HC conditions, respectively. The initial cell concentration was 50 cells mL^–1^ and the cell densities were maintained within a range of ∼4.0 × 10^4^ to 5.0 × 10^5^ cells mL^–1^ at the time of dilution. The cultures were inoculated every 5–7 days to restore the cell density to the initial level (i.e., batch growth cycle) with fresh medium equilibrated with the corresponding target CO_2_ levels. To main a stable carbonate chemistry over each batch growth cycle (pH variations < 0.1 units), the cultures were maintained in closed polycarbonate bottles that were completely filled with culture medium to prevent head space gas exchange. Instead of analyzing the carbonate chemistry parameters on a weekly basis for a ∼400 days experiment, we measured the parameters before running the long-term selection experiments to ensure the semi-continuous culture approaches are reliable (Jin et al., 2013). In the pilot experiment, pH and dissolved inorganic carbon (DIC) were measured before and after the renewal of the medium in LC and HC cultures of *P. tricornutum*.

### Lipid Extraction

At the end of the long-term selection experiment, six replicate samples (*n* = 6) of the HC- and LC-selected cells were collected in the middle of the photoperiod, centrifuged (8,000 *g*, 10 min), flash frozen in liquid nitrogen, and stored at −80°C until further analysis. The pellets of freeze-dried cells were placed into a glass tube with a Teflon lined cap, and extracted in 0.75 mL methanol. Then, 2.5 mL of methyl tert-butyl ether (MTBE) was added and the mixture was incubated in a shaker at room temperature for 1 h. Phase separation was induced by adding 0.625 mL of UHPLC -grade water. After 10 min of incubation at room temperature, the sample was centrifuged at 1,000 *g* for 10 min. The upper organic phase was collected, while the lower phase was re-extracted with 1 mL of solvent mixture [MTBE/methanol/water (10:3:2.5, v/v/v)] and the resulting upper phase was collected again. The combined upper phases were dried with a Termovap sample concentrator (Ecom, Czechia). To speed up sample drying, 100 μL of MS-grade methanol was added to the upper phase after 25 min of centrifugation. Extracted lipids were dissolved in 100 μL CHCl_3_/ methanol/water (60:30:4.5, v/v/v) for storage until further analysis using liquid chromatography with tandem-mass spectrometry (LC-MS/MS).

### Liquid Chromatography With Tandem-Mass Spectrometry Analysis

Liquid chromatography with tandem-mass spectrometry analysis was performed using a Thermo Vanquish^TM^ UHPLC (ThermoFisher Scientific). Samples were injected into a Thermo Accucore C_30_ column using a 20-min linear gradient at a flow rate of 0.35 mL min^–1^. The column temperature was set at 40°C. Mobile phase buffer A was acetonitrile / water (6/4) with 10 mM ammonium acetate and 0.1% formic acid, whereas buffer B was acetonitrile/isopropanol (1/9) with 10 mM ammonium acetate and 0.1% formic acid. The solvent gradient was set as follows: 30% B, initial; 43% B, 8 min; 50% B, 8.1 min; 70% B, 17 min; 99% B, 24 min; 30% B, 27.1 min; 30% B, 31 min. The MS condition was set as follows: Q-Exactive mass series spectrometer was selected in the m/z 114-1700 scanning range, and the MS/MS scan was used for a data-dependent full scan. The Q-Exactive mass series spectrometer was operated in negative polarity mode with a spray voltage of 3 kV, capillary temperature of 350°C, sheath gas flow of 20 arbitrary units and auxiliary gas flow of five arbitrary units.

### Lipid Identification

The raw data files generated by the HPLC-MS/MS were processed using the software Compound Discoverer 3.0 (CD3.0, Thermo Fisher) to perform peak alignment, peak picking, and quantitation for each metabolite. The main parameters were set as follows: retention time tolerance: 0.2 min, actual mass tolerance: 5 ppm, signal intensity tolerance: 30%, signal/noise ratio: 3, and minimum intensity: 100,000. Peak intensities were normalized to the total spectral intensity. The normalized data were used to predict the molecular formula based on additive ions, molecular ion peaks and fragment ions. Peaks were matched with the databases LIPID MAPS^1^ and LipidBlast to obtain accurate qualitative and relative quantitative results. Then the identified metabolites were annotated using the Human Metabolome Database (HMDB)^2^ and LIPID MAPS database.

### Quality Evaluation of Lipid Metabolites Data

Quality control samples (QCs) were obtained by collecting an equal amount of mixture from each replicate sample. The consistency analysis was performed between QCs and our culture samples. Before the analysis, three QCs were used to stabilize the analysis system and to remove the acquired data before data processing. All QCs were used to monitor the robustness of sample preparation and the stability of instrumental analysis by analyzing batch random inserts. During the whole instrumental analysis process, all samples were analyzed randomly to avoid inter-batch differences (Wang et al., 2015). In order to evaluate the overfitting of the model, 200 permutation tests were performed in the partial least squares discriminant analysis (PLS-DA) model (Broadhurst and Kell, 2006).

### Statistical Analysis

After metabolic information collection and data pre-processing, the resulting matrix was imported into the software metaX (Wen et al., 2017) for unsupervised principal component analysis (PCA) and supervised PLS-DA (Roede et al., 2014). Identification of metabolites has a variable importance in the projection (VIP) graphs (99% confidence) (Roede et al., 2014). For each multivariate model, the calculated R^2^ value reflects the goodness of fit. The parameter Q2 in the PLS-DA represents the predictive ability of the model (Roede et al., 2014). A Q2 value close to 0.5 reflects a good model. We applied univariate analysis (*t*-test) to calculate the statistical significance (*p*-value). Metabolites with VIP > 1, *p* < 0.05 and fold change (FC) ≥ 2 or FC ≤ 0.5 were considered to be differential metabolites. Volcano plots were used to filter metabolites of interest, based on Log_2_ (FC) and -log_10_ (*p*-value) of metabolites. For clustering heat maps, the data were normalized using z-scores of the intensity areas of differential metabolites, and plotted using the *pheatmap* package in R (R version R.3.6.1) and TBtools (Chen et al., 2020). The correlations between differential metabolites were analyzed using the cor() function in R (method = Pearson). Statistical significances of correlation between differential metabolites were calculated by the function *cor.mtest*() in R. *P*-values < 0.05 were considered as statistically significant, and the correlation plots were generated using the *corrplot* package in R. The Kyoto Encyclopedia of Genes and Genome (KEGG) database was used for enrichment analysis and pathway analysis of differential metabolites. Chi-square test was used to test the differences in lipid metabolite compositions between long-term HC- and LC-selected cells.

## Results

### Sample Quality Control

The Pearson correlation coefficient between QC samples based on the peak area value found high correlation coefficients (∼0.99) of QC samples, which indicated a stability of the whole detection process and a high data quality (Supplementary Figure 1).

### Overall Metabolites Description

We identified a total of 476 lipid metabolites in long-term HC- and LC-selected *P. tricornutum* cells (Supplementary Table 1). At the lipid category level, the majority of lipid metabolites were glycerophospholipids (GP) (49%), followed by glycerolipids (GL) (29%). Other lipid categories, such as sphingolipids (SP) and fatty acyls (FA), contributed relatively small proportions (SP: 15%; FA: 6%) in the detected lipid metabolites. At the lipid class level, the main lipid classes identified in long-term HC- and LC-selected *P. tricornutum* cells were glycosyldiradylglycerols, accounting for 27% of total lipid metabolites (Supplementary Table 1). Other identified lipid classes, such as glycerophosphoglycerols, glycerophosphocholines, and ceramides, represented 19, 15, and 13% of the total lipid metabolite pool (Supplementary Table 1).

### Changes in Lipid Metabolites Between Long-Term HC- and LC-Selected Cells

The principal component analysis (PCA) of total lipid metabolites showed that PC1 and PC2 explain 43.4 and 31.1% of variation, respectively (Supplementary Figure 2). Although there were some variations among the six replicate samples due to the nature of the lipidomics analysis, our results showed there was a satisfactory separation of lipid metabolites between long-term HC- and LC-selected cells (Supplementary Figure 2). This discrimination was further evidenced by the PLS-DA scores plot (Supplementary Figure 3).

**FIGURE 1:**
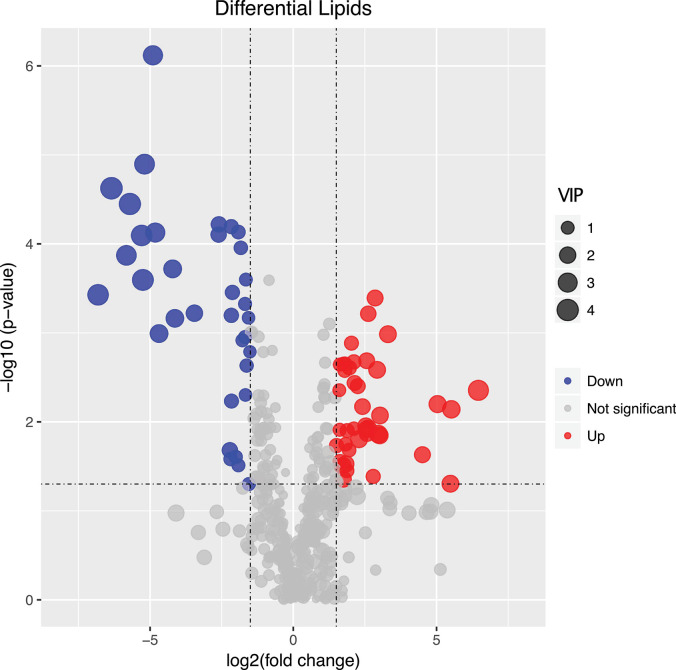
Volcano plot displaying the differentially regulated lipid metabolites between long-term low (400 μatm, LC) and high (1,000 μatm, HC) CO_2_ selected *Phaeodactylum tricornutum* cells. The dots represented in blue (down-regulated) and red (up-regulated) are differentially regulated lipid metabolites with > 2-fold change and a *p*-value of <0.05. VIP value represents the importance projection value of the metabolite obtained from the PLS-DA model.

**FIGURE 2:**
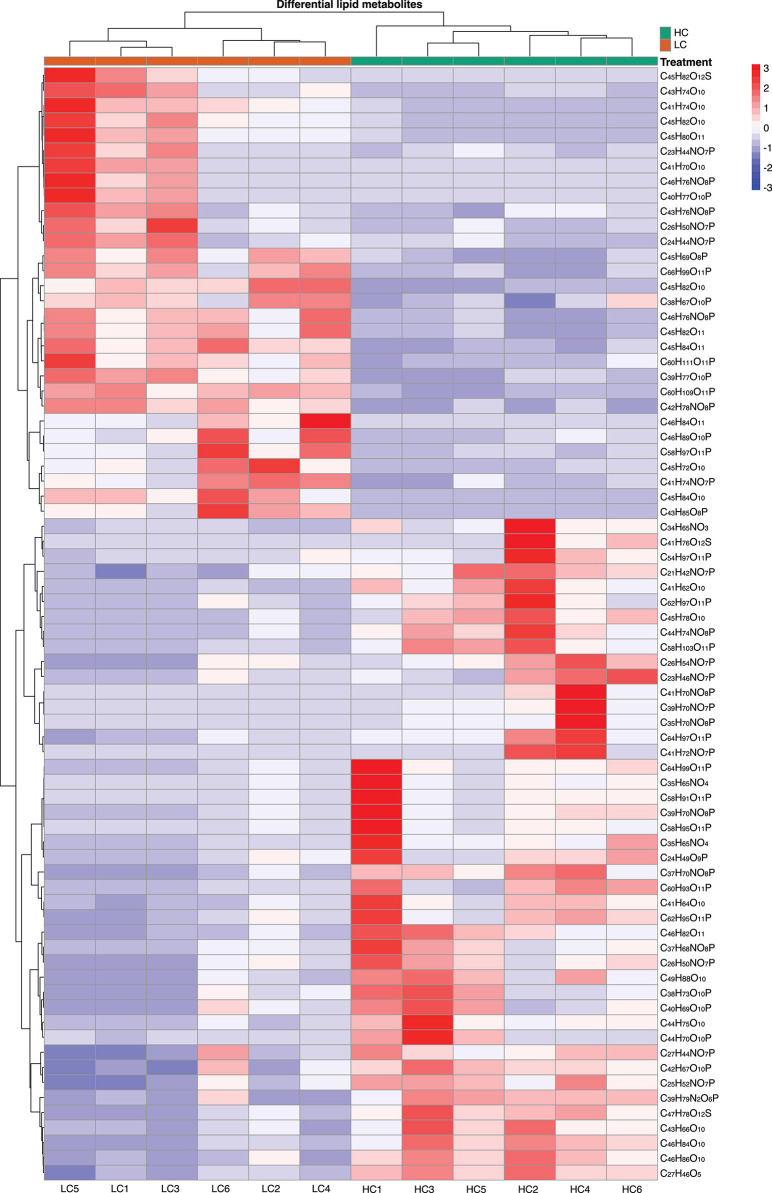
Heatmap displaying the log_2_-fold change in lipid metabolite between low (400 μatm, LC) and high (1,000 μatm, HC) CO_2_ selected *Phaeodactylum tricornutum* cells.

We found that of the identified 476 lipid metabolites, 74 significantly regulated different between long-term HC- and LC-selected cells (Table 1). Of those 74 lipid metabolites, 44 were significantly up-regulated, while the remaining 30 were significantly down-regulated in the long-term HC-selected cells compared to those under LC-selection (Table 1 and Figures 1, 2). Overall, the lipid classes MGDG (19%), HBMP (18%), PE (12%), and PG (12%) dominated in these 74 differentially regulated lipid metabolites (Table 1). However, the frequency of lipid classes differed significantly between down- and up-regulated lipid metabolites (χ^2^ = 65.317, *p* < 0.001, df = 16, *n* = 74) (Table 1). Specifically, MGDG (43%) was the most abundant in the down-regulated lipid metabolites, while HBMP (23%) and PE (20%) dominated in the up-regulated lipids (Table 1). We also found that the majority of the differently regulated PG lipids (seven out of nine) were up-regulated in long-term high CO_2_ selected cells (Table 1).

**TABLE 1:** Lipid metabolites that showed greater than twofold alterations in abundance in *Phaeodactylum tricornutum* selected under low (400 μam, ambient CO_2_ condition) or high CO_2_ (1,000 μam, projected year 2100 high CO_2_ according to high emission scenario RCP 8.5) for nearly 400 days.

**Annotation**	**Lipid class**	**RT**	**MW**	**Formula**	**Log_2_FC**	***p*-value**	**VIP**	**Change**
MGDG (37:1)	Glycosyldiradylglycerols	19.902	844.62783	C_46_H_86_O_10_	−4.90	<0.001	3.02	Down
GlcAG (37:2)	Glycosyldiradylglycerols	17.251	810.58572	C_46_H_82_O_11_	−5.19	<0.001	3.28	Down
MGDG (36:1)	Glycosyldiradylglycerols	19.376	830.61168	C_45_H_84_O_10_	−6.35	<0.001	4.06	Down
MGDG (36:2)	Glycosyldiradylglycerols	17.84	828.5959	C_45_H_82_O_10_	−5.70	<0.001	3.92	Down
MGDG (34:4)	Glycosyldiradylglycerols	14.714	796.5333	C_43_H_74_O_10_	−1.92	<0.001	1.19	Down
MGDG (36:2)	Glycosyldiradylglycerols	17.835	842.6116	C_45_H_82_O_10_	−4.82	<0.001	2.96	Down
GlcAG (37:1)	Glycosyldiacylglycerols	18.738	812.60128	C_46_H_84_O_11_	−5.29	<0.001	3.61	Down
MGDG (32:4)	Glycosyldiradylglycerols	13.289	768.50235	C_41_H_70_O_10_	−1.83	<0.001	1.14	Down
GlcAG (36:2)	Glycosyldiradylglycerols	16.612	796.5699	C_45_H_80_O_11_	−5.83	<0.001	3.22	Down
MGDG (37:2)	Glycosyldiradylglycerols	18.469	842.61183	C_46_H_84_O_10_	−4.21	<0.001	2.48	Down
SQDG (36:2)	Glycosyldiradylglycerols	15.457	846.55236	C_45_H_82_O_12_S	−5.25	<0.001	3.76	Down
GlcAG (36:1)	Glycosyldiradylglycerols	18.154	798.5855	C_45_H_82_O_11_	−6.81	<0.001	3.63	Down
SQDG (32:1)	Glycosyldiradylglycerols	14.757	792.50538	C_41_H_76_O_12_S	2.86	<0.001	1.85	Up
MGDG (36:7)	Glycosyldiradylglycerols	12.438	818.51756	C_45_H_72_O_10_	−1.68	<0.001	1.04	Down
MGDG (40:3)	Glycosyldiradylglycerols	20.767	882.6431	C_49_H_8_O_10_	2.62	0.001	1.71	Up
MGDG (36:4)	Glycosyldiradylglycerols	16.512	1,649.13028	C_45_ H_78_O_10_	−2.16	0.001	1.36	Down
MGDG (32:8)	Glycosyldiradylglycerols	7.549	760.44013	C_41_H_62_O_10_	−4.13	0.001	2.42	Down
GlcAG (36:0)	Glycosyldiacylglycerols	19.629	800.60171	C_45_H_84_O_11_	−4.69	0.001	2.48	Down
MGDG (32:2)	Glycosyldiradylglycerols	14.717	786.54897	C_41_H_74_O_10_	−1.68	0.001	1.03	Down
MGDG (32:7)	Glycosyldiradylglycerols	9.127	776.47078	C_41_H_64_O_10_	−1.63	0.002	1.09	Down
SQDG (38:6)	Glycosyldiradylglycerols	13.527	866.52094	C_47_H_78_O_12_S	2.30	0.016	2.23	Up
MGDG (34:8)	Glycosyldiradylglycerols	9.946	788.47124	C_43_H_66_O_10_	−2.21	0.021	1.66	Down
PG (33:0)	Glycerophosphoglycerols	16.299	1,473.05044	C_39_H_77_O_10_P	−2.60	<0.001	1.67	Down
HBMP (54:3)	Glycerophosphoglycerols	22.134	1,038.78583	C_60_ H_111_O_11_P	−2.13	<0.001	1.34	Down
PG (32:1)	Glycerophosphoglycerols	15.427	720.49411	C_38_H_73_O_10_P	2.03	0.001	1.28	Up
PG (34:5)	Glycerophosphoglycerols	12.299	740.46268	C_40_H_69_O_10_P	2.56	0.002	1.82	Up
HBMP (48:4)	Glycerophosphoglycerols	19.577	952.6745	C_54_ H_97_O_11_P	2.11	0.002	1.37	Up
PG (40:1)	Glycerophosphoglycerols	21.197	832.61914	C_46_H_89_O_10_P	2.93	0.003	2.14	Up
PG (38:5)	Glycerophosphoglycerols	14.093	796.51613	C_44_H_77_O_10_P	1.80	0.003	1.28	Up
PG (32:4)	Glycerophosphoglycerols	11.828	714.44694	C_38_H_67_O_10_P	2.14	0.004	1.53	Up
HBMP (60:15)	Glycerophosphoglycerols	17.82	1,098.69968	C_66_H_99_O_11_P	−2.16	0.006	1.27	Down
HBMP (58:14)	Glycerophosphoglycerols	17.099	1,072.6759	C_64_H_97_O_11_P	3.03	0.008	2.12	Up
HBMP (54:12)	Glycerophosphoglycerols	16.066	1,020.64492	C_60_H_93_O_11_P	2.53	0.011	1.95	Up
HBMP (52:9)	Glycerophosphoglycerols	17.465	998.66072	C_58_H_95_O_11_P	2.62	0.012	2.03	Up
HBMP (58:13)	Glycerophosphoglycerols	17.506	1,074.69166	C_64_H_99_O_11_P	2.55	0.012	2.00	Up
HBMP (52:8)	Glycerophosphoglycerols	18.744	1,000.67935	C_58_H_97_O_11_P	1.88	0.013	1.30	Up
HBMP (52:11)	Glycerophosphoglycerols	15.691	994.62916	C_58_H_91_O_11_P	2.58	0.014	1.63	Up
HBMP (56:13)	Glycerophosphoglycerols	16.549	1,046.6605	C_62_H_95_O_11_P	2.97	0.014	2.46	Up
PG (36:8)	Glycerophosphoglycerols	8.839	762.4466	C_42_H_67_O_10_P	3.02	0.014	2.15	Up
HBMP (52:5)	Glycerophosphoglycerols	20.951	1,006.72378	C_58_H_103_O_11_P	1.63	0.028	1.26	Up
LPG (18:0)	Glycerophosphoglycerols	3.953	512.31077	C_24_H_49_O_9_P	1.29	0.029	1.21	Up
HBMP (56:12)	Glycerophosphoglycerols	17.205	1,048.67647	C_62_H_97_O_11_P	1.65	0.032	1.07	Up
PG (38:6)	Glycerophosphoglycerols	12.303	794.50964	C_44_H_75_O_10_P	1.87	0.035	1.26	Up
PG (34:1)	Glycerophosphoglycerols	16.164	748.53171	C_40_H_77_O_10_P	−1.25	0.042	1.00	Down
HBMP (54:4)	Glycerophosphoglycerols	21.665	1,036.7705	C_60_H_109_O_11_P	−1.55	0.050	1.03	Down
PC (36:7)	Glycerophosphocholines	10.633	821.51167	C_44_H_74_NO_8_P	−2.16	<0.001	1.33	Down
LPC (17:0)	Glycerophosphocholines	3.388	555.35194	C_25_H_52_NO_7_P	2.42	0.007	1.77	Up
LPC (18:2)	Glycerophosphocholines	2.043	579.35316	C_26_H_50_NO_7_P	1.83	0.018	1.17	Up
LPC (18:0)	Glycerophosphocholines	4.491	569.36864	C_26_H_54_NO_7_P	1.51	0.018	1.33	Up
PC (34:3)	Glycerophosphocholines	14.846	801.55194	C_42_H_78_NO_8_P	−2.00	0.025	1.04	Down
PC (38:8)	Glycerophosphocholines	12.205	847.53582	C_46_H_76_NO_8_P	−2.19	0.026	1.13	Down
PC (38:7)	Glycerophosphocholines	13.058	849.55163	C_46_H_78_NO_8_P	−1.92	0.031	1.01	Down
LPC (16:3)	Glycerophosphocholines	1.229	535.2892	C_24_H_44_NO_7_P	2.79	0.041	1.34	Up
LPC (18:2)	Glycerophosphocholines	2.064	565.33693	C_26_H_50_NO_7_P	1.50	0.047	1.27	Up
PE (36:6)	Glycerophosphoethanolamines	15.429	721.49724	C_41_H_72_NO_7_P	1.80	0.002	1.21	Up
PE (36:6)	Glycerophosphoethanolamines	13.041	735.48352	C_41_H_70_NO_8_P	1.75	0.002	1.11	Up
PE (38:5)	Glycerophosphoethanolamines	15.889	765.53126	C_43_H_76_NO_8_P	1.63	0.002	1.04	Up
PE (32:3)	Glycerophosphoethanolamines	12.794	685.46822	C_37_H_68_NO_8_P	1.96	0.002	1.31	Up
LPE (16:1)	Glycerophosphoethanolamines	2.388	451.26933	C_21_H_42_NO_7_P	2.25	0.004	1.54	Up
PE (36:5)	Glycerophosphoethanolamines	15.84	723.52023	C_41_H_74_NO_7_P	6.46	0.004	3.42	Up
PE (34:4)	Glycerophosphoethanolamines	14.065	711.48427	C_39_H_70_NO_8_P	1.62	0.012	1.03	Up
PE (32:2)	Glycerophosphoethanolamines	14.05	687.4837	C_37_H_70_NO_8_P	1.95	0.021	1.19	Up
PE (30:0)	Glycerophosphoethanolamines	14.832	663.48381	C_35_H_70_NO_8_P	4.51	0.023	1.86	Up
LPE (18:2)	Glycerophosphoethanolamines	2.181	477.2847	C_23_H_44_NO_7_P	1.83	0.030	1.95	Up
LPE (18:1)	Glycerophosphoethanolamines	2.949	479.30054	C_23_H_46_NO_7_P	1.74	0.031	1.76	Up
PE (34:5)	Glycerophosphoethanolamines	13.344	695.4817	C_39_H_70_NO_7_P	1.43	0.035	1.25	Up
LPE (22:6)	Glycerophosphoethanolamines	1.986	525.28436	C_27_H_44_NO_7_P	1.75	0.044	1.81	Up
Cer-AS (35:3)	Ceramides	14.958	609.49647	C_35_H_65_NO_4_	5.03	0.006	2.24	Up
Cer-NP (35:3)	Ceramides	14.944	563.49096	C_35_H_65_NO_4_	5.52	0.007	2.38	Up
Cer-NS (34:2)	Ceramides	15.5	535.49586	C_34_H_65_NO_3_	5.49	0.050	2.18	Up
3α,7α,12α-trihydroxy-5β-cholestan-26-oic acid	Bile acids and derivatives	4.746	450.33401	C_27_H_46_O_5_	2.12	0.012	1.14	Up
PetOH (40:10)	Other Glycerophospholipids	10.633	768.48079	C_45_H_69_O_8_P	−2.61	<0.001	1.65	Down
1-P-3-S-2-P	Glycerophosphates	10.68	760.59645	C_43_H_85_O_8_P	−3.45	0.001	2.01	Down
SM (34:1)	Phosphosphingolipids	14.98	748.57318	C_39_H_79_N_2_O_6_P	3.30	0.001	2.16	Up

*RT, HPLC-MS/MS retention time (min); MW, molecular weight; VIP, variable importance in the projection.*

### Correlations of Differently Regulated Metabolites

**FIGURE 3:**
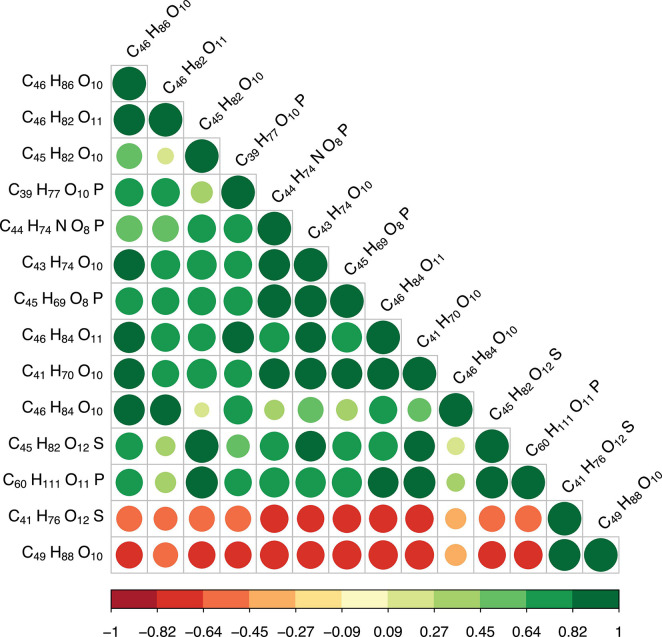
Pearson correlations between differential metabolites (top 20). The size of the circle represents the magnitude of correlation. Green circles indicate positive correlations, red negative correlations. The value of the correlation coefficient (*r*) is shown in the color bar below the graphs.

We further explored the correlations between differently regulated metabolites. Our results showed that MGDG (37:1, C_46_H_86_O_10_) were positively correlated with MGDG (34:4, C_43_H_74_O_10_), MGDG (32:4, C_46_H_86_O_10_), MGDG (37:2, C_46_H_84_O_10_) and GlcADG (37:1, C_46_H_82_O_11_) [Pearson correlation coefficients (*r*): 0.70–0.86] (Figure 3). PC (36:7, C_44_H_74_NO_8_P) were positively correlated with three MGDGs (36:2, C_45_H_82_O_10_; 34:4, C_43_H_74_O_10_, 32:4, C_41_H_70_O_10_) (*r*: 0.70–0.87). PC (36:7, C_44_H_74_NO_8_P) was positively correlated with GlcADG (37:1, C_46_H_84_O_11_) (*r*: 0.80), SQDG (36:2, C_45_H_82_O_12_S) (*r*: 0.74), HBMP (54:3, C_60_H_111_O_11_P) (*r*: 0.80), PG (33:0, C_39_H_77_O_10_P) (*r*: 0.73), and PEtOH (40:10, C_45_H_69_O_81_P) (*r*: 0.99) (Figure 3). In contrast, PC was negatively correlated with SQDG (32:1, C_41_H_76_O_12_S) (*r*: −0.70) and MGDGs (40:3, C_49_H_88_O_10_) (*r*: −0.73) (Figure 3). There were also pronounced negative correlations of two metabolites (MGDG, 40:3, C_49_H_88_O_10_; SQDG, 32:1, C_41_H_76_O_12_S) with the other metabolites presented in Figure 3 [e.g., SQDG (C_41_H_76_O_12_S) with PG (C_39_H_77_O_10_P), *r*: −0.6]. In summary, the up- and down-regulations of the metabolites were tightly coupled either by negative or positive correlations.

## Discussion

Our results show that after 400 days (corresponding to almost 900 generations) of long-term high CO_2_ selection, the marine diatom species *P. tricornutum* revealed significant changes in lipid metabolisms. One of the most apparent findings of the present study is that MGDG, the main glycerolipid in non-plastid membranes, was significantly down-regulated in long-term LC selected cells. Our results are in agreement with the results of a previous short-term (6 h) study with the green algae species *Chlamydomonas reinhardtii* (Abreu et al., 2020). MGDG is a hexagonal II phase polar membrane lipid, whose role is to give the membrane a high internal lateral pressure among the fatty acyl chains and a pressure on the membrane proteins (Kruijff, 1997). Due to its non-bilayer lipid properties, different ratios of MGDG to other phase properties lipids may affect the lateral pressure on membrane proteins (Kruijff, 1997). For instance, it has been widely reported that a lower ratio of DGDG (a lamellar phase lipid) to MGDG reflects increased sensitivities of microalgae to various abiotic stresses (e.g., low temperature, CO_2_ limitation, salt stress, nitrogen starvation) (Du et al., 2018; Liu et al., 2019). Since no significant changes of DGDG were detected in the present study, the down-regulations of MGDG in long-term HC selected cells resulted in an increased ratio of DGDG:MGDG. Therefore, our results suggest that long-term high CO_2_ selection may be not stressful for *P. tricornutum*.

It is also worth noting that the down-regulations of MGDG would result in an increased conductivity of the thylakoid and in an increase of luminal pH, causing the activity of violaxanthin de-epoxidase to decrease (Aronsson et al., 2008). In addition, the down-regulations of MGDG are also likely to decrease the violaxanthin availability from the membrane for violaxanthin de-epoxidase (Schaller et al., 2010). Consequently, such a decrease in de-epoxidase would lead to a low efficiency of the violaxanthin-zeaxanthin interconversion, which is a key process of the xanthophyll cycle, a crucial regulatory component for energy dissipation in diatoms (Miloslavina et al., 2009; Goss and Jakob, 2010; Lepetit et al., 2017). This is evidenced by previous studies, in which diatoms in high CO_2_ conditions exhibited higher non-photochemical quenching (NPQ) (i.e., a lower energy dissipation capacity) (e.g., Gao et al., 2012; Wu et al., 2017; Li et al., 2019). These findings were further supported by our recent investigations, in which we found three proteins, namely the precursor protein of zeaxanthin epoxidase (ZEP2), zeaxanthin epoxidase (ZEP1) and violaxanthin de-epoxidase (VDE), were significantly down-regulated in HC selected cells (data not shown). To summarize, our results suggest that the MGDG down-regulation in long-term HC selected *P. tricornutum* cells is likely an adaptation strategy to cope with the long-term high CO_2_ conditions.

PE is one of the major phospholipids (phosphoglycerides) in most algae species (Guschina and Harwood, 2009). Very long chain polyunsaturated fatty acid can be incorporated into PE by acyltransferase reactions of the Kennedy pathway (Sayanova et al., 2017; Li-Beisson et al., 2019). The production of PE by algae was recognized as a response to abiotic stresses, such as light, temperature and nutrient. For instance, it was evidenced that the content of PE significantly increased with increasing temperature (Thompson, 1996). Both dark exposure and phosphate starvation were found to lead to a decrease of PE content in microalgae species (McLarnon-Riches et al., 1998; Khozin-Goldberg and Cohen, 2006). For the response of PE to high CO_2_, the degree of unsaturation of PE was reported to decrease in response to high CO_2_ by decreasing the contents of C18:3 at the *sn*-2 position of PE (Sato et al., 2003). Our results indicated that rather than the unsaturation of PE, the concentration of PE was significantly enhanced under long-term high CO_2_ selection.

**FIGURE 4:**
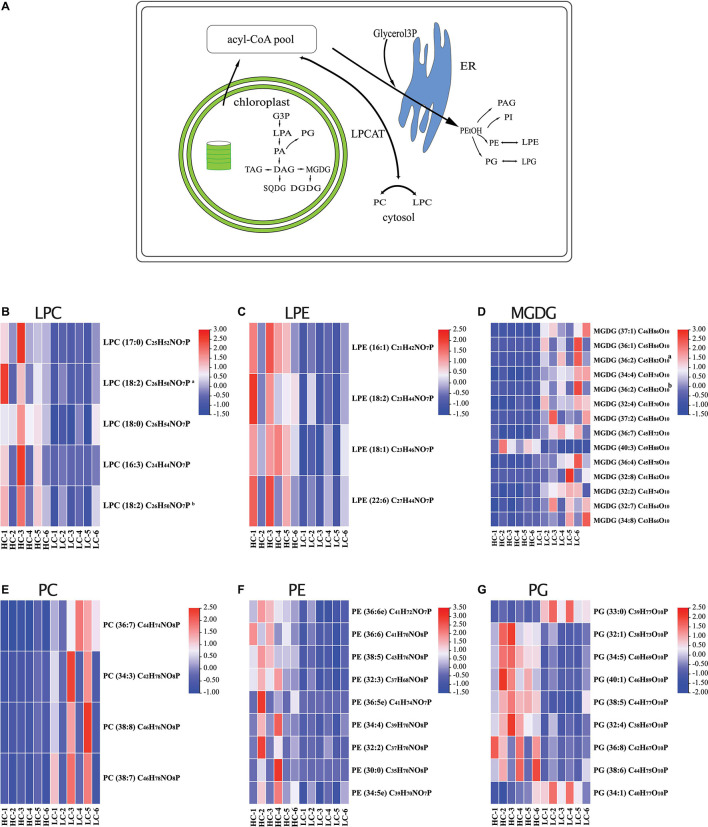
**(A)** Model of lipid metabolism changes in *Phaeodactylum tricornutum* after long-term high CO_2_ selection. The heatmaps display the log_2_-fold changes in lysophosphatidylcholine [LPC, **(B)**], lysophosphatidylethanolamine LPE **(C)**, monogalactosyldiacylglycerol [MGDG, **(D)**], PC, phosphatidylcholine [PC, **(E)**], phosphatidylethanolamine [PE, **(F)**], and phosphatidylglycerol [PG, **(G)**].

In addition to PE, the majority of the differently regulated PG lipids (seven out of nine) were up-regulated in long-term high CO_2_ selected cells (Figure 4). PG is the major phospholipid in chloroplasts, and it contains an uncommon *trans-*3-hexadecenoic acid (C16:1t), located exclusively at position *sn*-2 of the glycerol backbone in all eukaryotic photosynthetic organisms (Boudière et al., 2014; Figure 4). PG play an important role in the light harvesting complex trimerization process and in the functioning of the photosystem (Loll et al., 2007). Along with MGDG and PE, PG is also sensitive to various abiotic stressors. It is well recognized that the contents of PG in various algal species, including diatoms, decrease under nutrient starvation (Van Mooy et al., 2009; Abida et al., 2015; Wang et al., 2019), elevated temperatures (Feijão et al., 2017, 2020), and low pH conditions (Vítová et al., 2016). In the present study, we found that ∼80% of the significantly regulated PGs were up-regulated after long-term high CO_2_ selection (Figure 4). This may be due to adaptation driven by long-term high CO_2_ selection. While the content of PG may decrease after short-term high CO_2_ selection, its content may be partially or completely reversed over the long-term high CO_2_ selection period. Such an adaptive response has been evidenced in multiple previous studies (Lohbeck et al., 2012; Jin et al., 2020). For instance, the fatty acid and lipid contents of three diatoms partly or entirely recovered following a long-term exposure (∼2 years) to warming conditions (+4°C) (Jin et al., 2020). Thus, our results suggest a high potential of diatoms to adapt their lipid metabolites to long-term high CO_2_ selection.

It is known that an increase of SQDG could compensate the absence of PG in plastids (Jouhet et al., 2010; Boudière et al., 2014; Abida et al., 2015), thus the contents of SQDG and PG are expected to be negatively correlated, as shown in our study. Under stressful conditions, such as nutrient starvation, a PG-to-SQDG replacement was considered to be a ubiquitous phenomenon in photosynthetic organisms, enabling the preservation of an anionic lipid environment to the photosystems in the thylakoids (Boudière et al., 2014; Nakamura and Li-Beisson, 2016). Besides a PG-to-SQDG replacement, there was also a PC-to-DGTA replacement occurring in the diatom *P. tricornutum*, which was also considered to be a strategy to cope with environmental stresses (Abida et al., 2015). Since we observed multiple negative or positive correlations between different lipid metabolites (e.g., positive correlation between PC and PEtOH; negative correlations between MGDG and PC), our results suggest several more lipid remodeling mechanisms that have not been identified yet. We propose that the underlying mechanisms for lipid remodeling in response to various abiotic stressors in diatoms should be further investigated as part of future studies.

It is also recognized that the lipid metabolism of microalgae was regulated by some mineral elements such as silicon and calcium. For instance, lipid production in marine diatoms *Chaetoceros gracilis* and *Thalassiosira pseudonana* was reported to increase under silicon limitation conditions (Zendejas et al., 2012; Adams and Bugbee, 2014). Since high CO_2_ was reported to affect the biogenic silica cellular contents of diatoms (Xu et al., 2014), such a change induced by high CO_2_ is expected to regulate the lipid metabolism as well. Calcium (Ca^2+^), a secondary messenger, plays a crucial role in the signal transduction through the activation of various receptors associated with metabolic in coping with various environmental changes (Chen et al., 2014). Calcium can bind with calmodulin and then affect the energy metabolism by regulating the cyclic electron flow in photophosphorylation and respiratory oxidative phosphorylation in microalgae (Chen et al., 2015). It is known that an appropriate concentration of calcium can stimulate the activity of Acetyl-CoA carboxylase that catalyzes the conversion of acetyl-coenzyme A to malonyl-CoA during the fatty acid biosynthesis (Gorain et al., 2013). However, how would high CO_2_ interact with calcium to regulate the lipid modeling of diatoms (through energy metabolism or signal transduction) remains unknown. Hence, we recommend that the underlying mechanisms for lipid remodeling in response to high CO_2_ interacting with essential (or non-essential) elements in diatoms are warranted for further investigations.

In conclusion, our observations suggest that long-term high CO_2_ selection triggered substantial changes in lipid metabolites of the marine diatom *P. tricornutum* (Figure 4A). Some lipid metabolites (such as PGs) showed high adaptive potential to the selection. The tightly coupled regulations (positively or negatively correlated) of lipid metabolites reveal that the lipid remodeling is an organismal adaptation strategy of marine diatoms to ongoing ocean acidification. The composition and concentration of lipid are crucial for marine food quality, and their changes can be transferred to high trophic levels (Rossoll et al., 2012; Jin et al., 2015, 2020). Therefore, our results outline the importance of investigating the long-term responses of lipids of primary producers to prolonged ocean acidification conditions, and assessing the ecological consequences.

## Data Availability Statement

The original contributions presented in the study are included in the article/Supplementary Material, further inquiries can be directed to the corresponding author/s.

## Author Contributions

PJ and JX conceptualized the study. ZL, HL, JP, PL, QH, YG, and JZ acquired the data. PJ and FL performed the statistical analysis and generated the figures with JW and SO. PJ drafted the manuscript, SO contributed substantially to the editing of the manuscript. All authors discussed and approved the manuscript.

## Conflict of Interest

The authors declare that the research was conducted in the absence of any commercial or financial relationships that could be construed as a potential conflict of interest.

## Publisher’s Note

All claims expressed in this article are solely those of the authors and do not necessarily represent those of their affiliated organizations, or those of the publisher, the editors and the reviewers. Any product that may be evaluated in this article, or claim that may be made by its manufacturer, is not guaranteed or endorsed by the publisher.

## Footnotes

1
http://www.lipidmaps.org/

2
http://www.hmdb.ca/

## Supplementary Material

Supplementary Figure 1 Pearson correlations between Quality control samples (QCs).

Supplementary Figure 2 The principal component analysis (PCA) of total lipid metabolites.

Supplementary Figure 3 Score plot from partial least squares discriminant analysis (PLS-DA) of the lipids in low (400 μatm, LC) and high (1,000 μatm, HC) CO_2_ selected *Phaeodactylum tricornutum* cells. The PLS-DA showed a clear separation between the HC and LC selected cells with acceptable goodness of fit (*R*^2^ = 0.97) and predictive power (*Q*^2^ = 0.91).
