# Supplementary figures and images for: Lipid Remodeling Reveals the Adaptations of a Marine Diatom to Ocean Acidification
Source: Front Microbiol. 2021 Oct 14;12:748445. doi: 10.3389/fmicb.2021.748445 (PMC8551959; doi:10.3389/fmicb.2021.748445)

Pearson correlation between QC samples

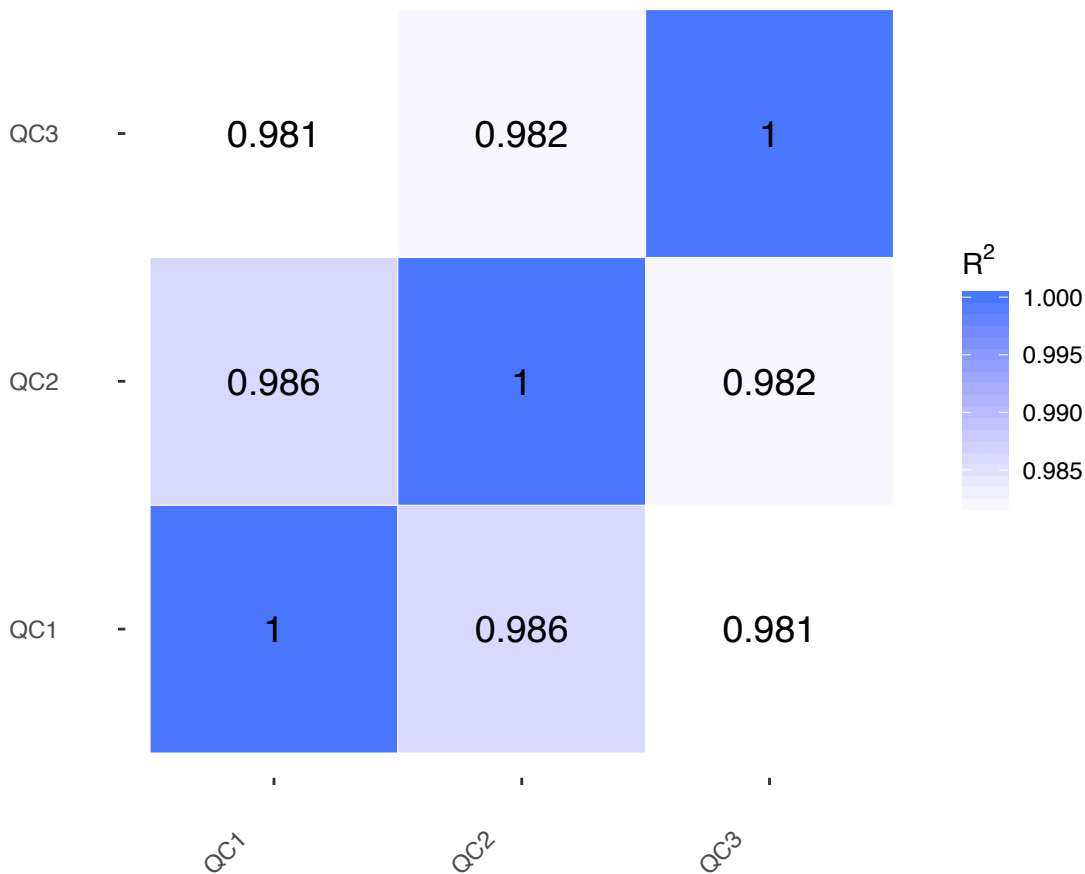

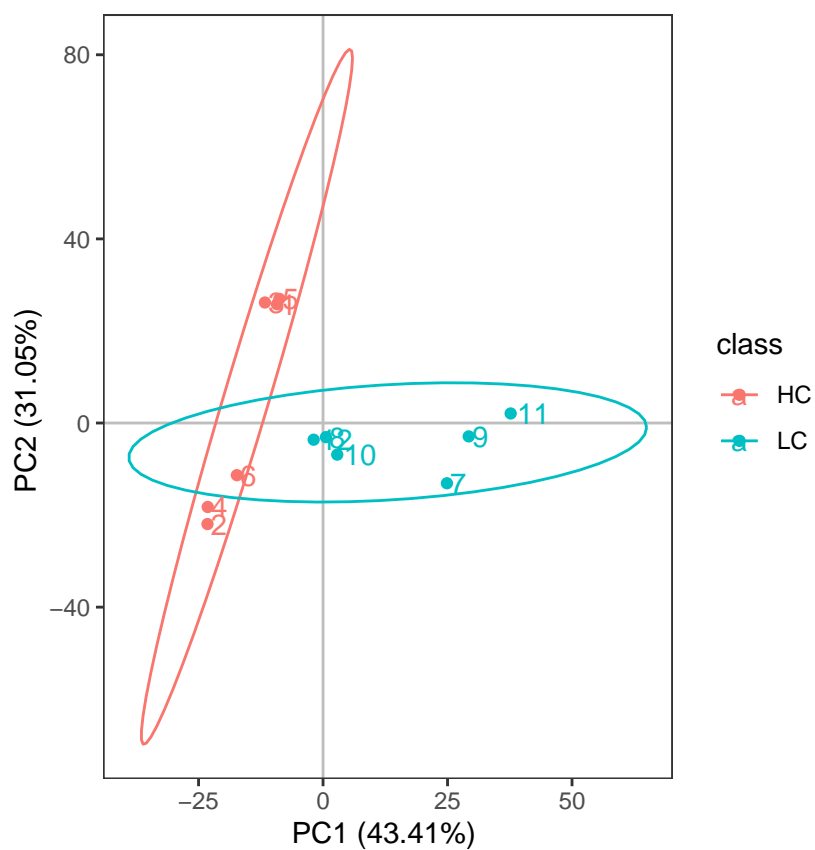

class    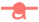 HC    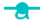 LC

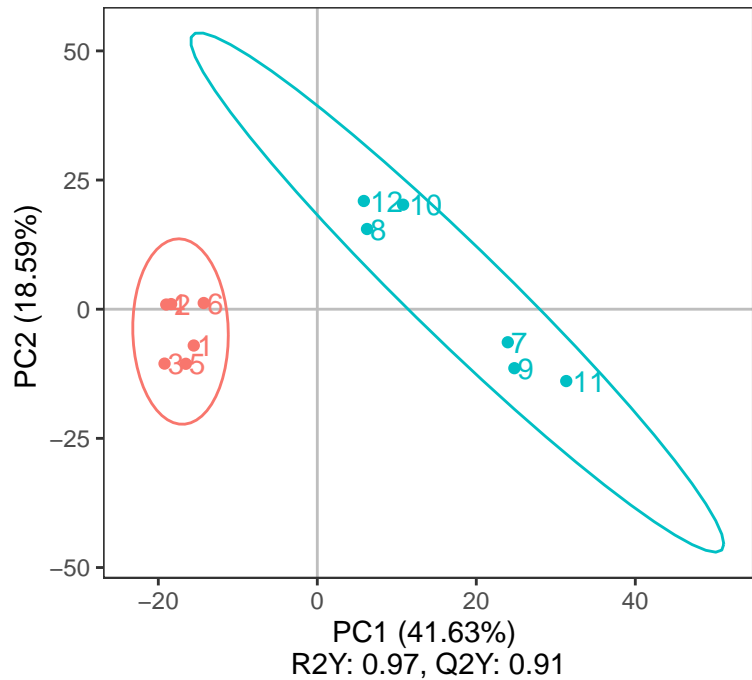

Supplement: Supplementary Figure 1 — Pearson correlations between Quality control samples (QCs). [file Presentation_1.pdf]
